# Shape Dependent EMA Model of Nanostructured Anisotropic Materials

**DOI:** 10.3390/nano9101380

**Published:** 2019-09-26

**Authors:** Petr Otipka, Jaroslav Vlček

**Affiliations:** 1 Department of Mathematics and Descriptive Geometry, Faculty of Mechanical Engineering, VSB—Technical University of Ostrava, 708 00 Ostrava-Poruba, Czech Republic; jaroslav.vlcek@vsb.cz; 2 Nanotechnology Centre, VSB—Technical University of Ostrava, 708 00 Ostrava-Poruba, Czech Republic

**Keywords:** biosensors, effective medium, nanoparticles, polarizability, SCD method, Green tensor

## Abstract

Heterogeneous nanostructures containing nanoparticles of various sizes and shapes have attracted significant attention in the development of nano-biosensors. Especially, plasmonic properties of such materials are advantageously exploited for the detection of biological and chemical substances. Since these media exhibit optical anisotropy, a valid homogenization procedure must be able to describe appropriately the relationship between the geometry of the inclusions and the nature of local field modes. We present a model approach for extension of the effective medium approximation (EMA) and its application to anisotropic nanostructures. The proposed model is based on a “strong-couple-dipole” (SCD) method including a volume-integral correction term in a Green tensor that enables to obtain more accurate representation of polarizability tensor. Derived depolarization factors for discs and bi-cone particles are compared with the early known shapes (spheroids, cylinders) and applied to nanostructures composed of the Fe or Au nanodots in polyacrylate.

## 1. Introduction

The development of novel nano-fabrication technologies has attracted significant attention because of the plasmonic properties of nanomaterials and the feasibility of exploiting them for the detection of biological and chemical substances [1]. Noble metal nanoparticles, such as gold and silver, exhibit unique optical resonance properties, and they have been proven to be useful for these applications [2,3,4]. Therefore, the immobilization of nanostructures with nanoparticles of various sizes and shapes is an important fabrication process for the development of nano-biosensors [5,6]. Increasing requirements of the accuracy and reliability of sensing devices can be advantageously solved by theoretical modelling [7,8]. A tentative design of composite nanostructures based on an appropriate theoretical model brings the possibility of pragmatic tuning of the required properties at a low-cost.

The applicability of effective medium approximation (EMA) is restricted by the size of the structures composing the mixture; sufficiently large to preserve locally their own electromagnetic behavior and small enough for the composite to appear homogeneous compared to the wavelength of the interacting radiation. Since the traditional approach for effective permittivity introduction used for isotropic media [9] is not satisfactory for such materials, models exploiting depolarization tensors have been developed [10,11], however, only for spheroidal inclusions. As the size and/or number of particle shapes should be explicitly incorporated within homogenization procedures, the deriving of corresponding depolarization tensors demands generalized methods, based, for example, on the Green electromagnetic tensor application [7,12]. If the nanoparticles are not extremely small, then the spatial extent of an associated Green tensor should not be neglected [9].

The article is organized as follows: The Maxwell-Garnett model of EMA and the basic principle of SCD (strong-couple-dipole) polarizability model are introduced in the next section in their generalized forms for homogeneous anisotropic nanoparticles. This theory is presented with an extension for disc- and cone-shaped inclusions because depolarization factors for nanoparticles of these shapes have not been referred to so far in detail. Further, in Section 2, there are also disclosed newly derived correction terms for cylindrical and conical particles. The third section contains, among other things, the application leading to the magneto-plasmonic (MO-SPR) response in the effective permittivity of Fe and Au nanoparticles deposited in polyacrylate. The importance of correction factors is also discussed in this section.

## 2. Methods

### 2.1. Effective Permittivity and Green Electromagnetic Tensor

If the electrostatic interaction between nanoparticles is not negligible, it should be taken into account by the generalized Maxwell-Garnett approach. It estimates the macroscopic response of the composite as an average effect of the dipole field induced in the host medium by different inclusions. This can be done by the Bragg-Pippard model of EMA [8] with a modification for a bi-anisotropic case [11,13]. Assuming volume fraction *f* of the metallic nanoparticles is in a host medium, effective permittivity tensor **ε***_ef_* can be written as

(1) $$\varepsilon_{ef}=\varepsilon_{h}+f(\varepsilon -\varepsilon_{h}){\left[fI+(1-f)v\;\alpha^{-1}(\varepsilon -\varepsilon_{h})\right]}^{-1}$$

The depolarization field is characterized through polarizability tensor **α** in the Equation (1) by wavelength λ (see wavenumber *k*_0_ = 2π/λ), as well as by the material properties of particles (permittivity tensor **ε**) and the permittivity of the host medium (**ε***_h_*). Its frequently used form [9,10],

$$\alpha =(\varepsilon -\varepsilon_{h}){\left[\varepsilon_{h}+L(\varepsilon -\varepsilon_{h})\right]}^{-1}\varepsilon_{h},$$

is expressed as a function of tensor **L** of depolarization factors, mirroring only the spheroidal particles of given volume *v*. In our version, we worked with Green tensor **G** that enabled us to also take in account other types of geometries of single particles.

In cases when the inclusions aligned with principal axes, the polarizability tensor can be expressed in the form [12]

(2) $$\alpha =v(\varepsilon -\varepsilon_{h}I){\left[I-k_{0}^{2}v\langle G\rangle (\varepsilon -\varepsilon_{h}I)\right]}^{-1}$$

where *k*_0_ = 2π/λ. Denoting the wavenumber $k=k_{0}\sqrt{\varepsilon_{h}}$, Green tensor **G** is defined as

(3) $$G(r,r_{0})=\left(I\;+\frac{1}{k^{2}}\nabla \times \nabla \right)g(r,r_{0})\;,\;g(r,r_{0})=\frac{1}{4\pi }\frac{e^{ik\left\|r-r_{0}\right\|}}{\left\|r-r_{0}\right\|}\;$$

where *g* is the free space Green function of the Helmholtz operator. Assuming an electrically small characteristic nanoparticle dimension, the tensor 〈G〉 averaged over a volume *v* with the unit outward normal vector ***n*** of its surface *S* can be written in the split form [14]

(4) $$v\langle G\rangle =\int_{v}G(r,r_{0})\;dv=\int_{v}\left(G-G_{s}\right)\;dv-\frac{1}{4\pi k^{2}}\int_{S}\frac{n⊗(r-r_{0})}{{\left\|r-r_{0}\right\|}^{3}}\;dS$$

with

(5) $$G_{s}=\frac{1}{4\pi k^{2}}\nabla ⊗\nabla \left(\frac{1}{\left\|r-r_{0}\right\|}\right)$$

### 2.2. Depolarizing Tensor

The surface integral

(6) $$L=\frac{1}{4\pi }\int_{S}\frac{n⊗(r-r_{0})}{{\left\|r-r_{0}\right\|}^{3}}\;dS$$

predominates in Equation (4), therefore, we meet its resolved form in numerous practical applications. Derivation of this term can be proved in several ways [4,15,16]. This integral does not depend on the volume, but rather on the geometrical shape of the particle. The results are well-known for current nanoparticle shapes [15,17,18] when these are axially and centrically symmetric.

**Figure 1:**
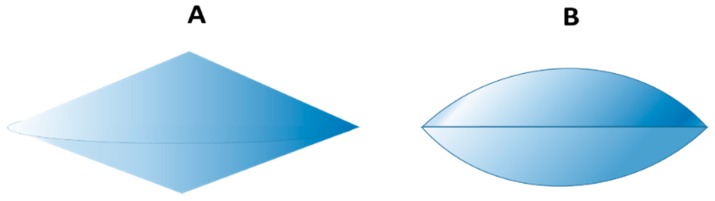
Nanoparticle shapes: (**A**) bi-cone, (**B**) disc.

Tensor **L** is symmetrical and real-valued; for appropriate orientation of principal axes, it becomes diagonal. Moreover, the trace Tr(**L**) = 1 over any sufficiently smooth surface (*S*). It means the third diagonal element of **L** is coupled with the other two; it is **L** = diag(*L*_⊥_, *L*_⊥_, 1 − 2*L*_⊥_) when the coordinate axis *x*_3_ is integrated with the particle axis. Besides the sphere (**L** = (1/3, 1/3, 1/3)), we mention at least the depolarizing factors of the cylinder and spheroid—see Table 1. In this work, we present newly obtained formulas for the disc and bi-cone (Figure 1).

**Figure 2:**
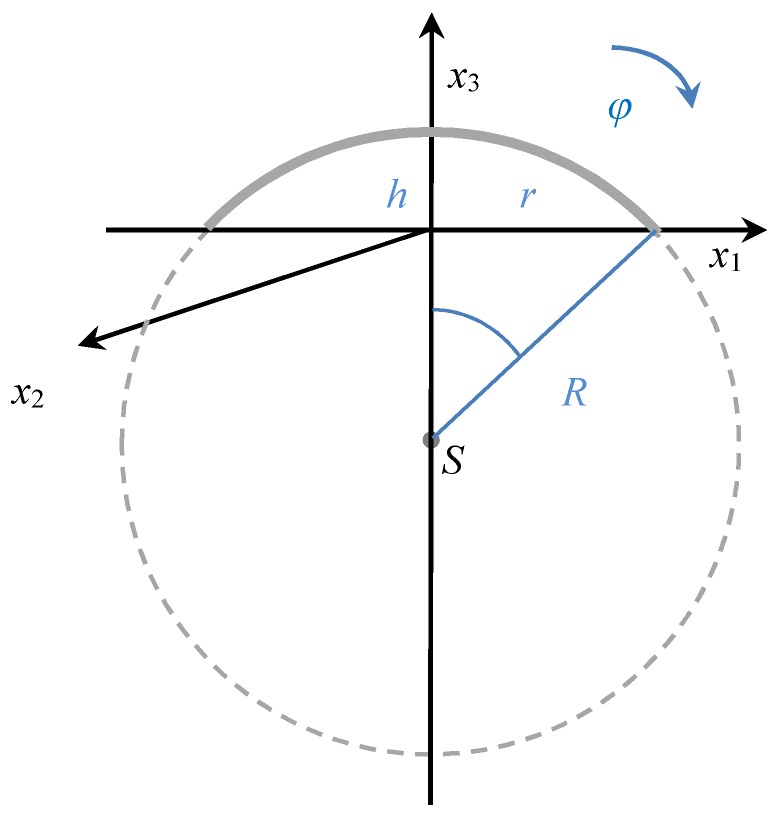
Disc–coordinate system and basic notation.

The main idea of the depolarizing factors derivation for the nano-disc consists in the following. Without loss of generality we identify the coordinate center with the center of the disc (Figure 2). The transform into spherical coordinates leads to the following expression of the radius vector ***r*** and normal vector ***n***:

(7)  r=(Rsinθcosφ,Rsinθsinφ,Rcosφ−R+h),   n=(sinθcosφ,sinθsinφ,cosφ)

where *φ* ∈ <0, 2π> and *θ* ∈<0, acos(1 − *h*/*R*)>. This step, among others, eliminates the singularity at point ***r*** = ***r***_0_ = ***o***.

After the integration of the surface integral (Equation (6)), the substitution *h*/*d* = *a* leads to the depolarizing factors in the form

(8) $$L_{⊥}=L_{11}=L_{22}=\frac{a\left(2a^{2}+3a+3\right)}{3{\left(1+a\right)}^{3}}\;,\;L_{∥}=L_{33}=-\frac{a^{3}-3a^{2}-3a-3}{3{\left(1+a\right)}^{3}}$$

In the case of the bi-cone, the coordinate center is located into the common center of platforms. Similar steps as in previous cases give the results presented in the third row of Table 1.

### 2.3. Correction Tensor

The role of volume averaged integral

(9) $$M=\int_{v}\left(G-G_{s}\right)dV$$

in Equation (4) is emphasized especially in the SCD applications, because it enables more adequate representation of nanoparticle polarizability. Note that volume *v* needs not be infinitesimal. The evaluation of this term also leads to the diagonal tensor whose elements for cylindrical and conical particle are newly derived.

Again, we suppose that the nanoparticle center of gravity is placed at the center of the coordinate system. Carrying out the tensor operations in Equations (3) and (5), the components of the Green tensor are expressed as

(10) $$G_{ij}=\frac{e^{ikr}}{4\pi k^{2}r^{3}}\left[\frac{x_{i}x_{j}}{r^{2}}\left(3-3ikr-k^{2}r^{2}\right)-\delta_{ij}\left(1-ikr-k^{2}r^{2}\right)\right]$$

(11) $$G_{s,ij}=\frac{1}{4\pi k^{2}r^{3}}\left[3\frac{x_{i}x_{j}}{r^{2}}-\delta_{ij}\right]$$

where δ*_ij_* denotes the Kronecker tensor. It can be easily shown that this term is zero-valued for spherical particles. In the case of the other symmetric particles, an appropriate coordinate transformation enables obtaining correction tensor elements in a closed form, keeping the assumption about sufficiently small particle dimensions compared with the wavelength of the acting electromagnetic field. The two derived correction terms are presented in Table 2.

## 3. Results and Discussion

The properties of a Green tensor related to various shapes of nanoparticles have an important influence on the effective permittivity defined by Equation (2). In any physical model, the effective permittivity is importantly influenced by the content of inclusions in the host medium, i.e., by filling factor *f*. Especially, if the metallic nanoparticles are exposed to an external magnetic field, then the induced anisotropy causes the presence of non-zero off-diagonal elements in the permittivity tensor. This effect is transferred into the polarizability tensor, and, subsequently, into the relative permittivity of the effective medium. In the presented examples, we assume the polar magneto-optical configuration, where the acting magnetic field is oriented perpendicular to the sample, i.e., parallel with the *x*_3_-axis (see Figure 2). Thus, the permittivity tensor has non-zero off-diagonal elements ε_12_ = −ε_21_ (=ε*_xy_*_, ef_).

**Figure 3:**
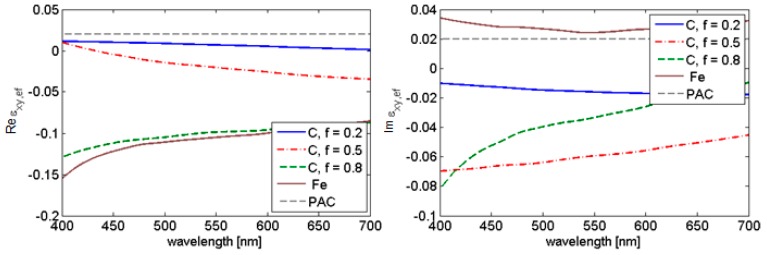
Off-diagonal components of effective permittivity for cylindrical Fe nanoparticles in polyacrylate (a = height/diameter = 10/5 nm, fill factor 0.2, 0.5, 0.8) compared to pure Fe [19] and polyacrylate.

The most conspicuous response is again observed for cylindrical nanoparticles in agreement with the previous result. This fact is demonstrated by tensor component ε*_xy_* in Figure 3 for the iron nanoparticles in polyacrylate by varying the fill factor. Optical functions of Fe and PAC (polyacrylate) are in [19,20], respectively.

**Figure 4:**
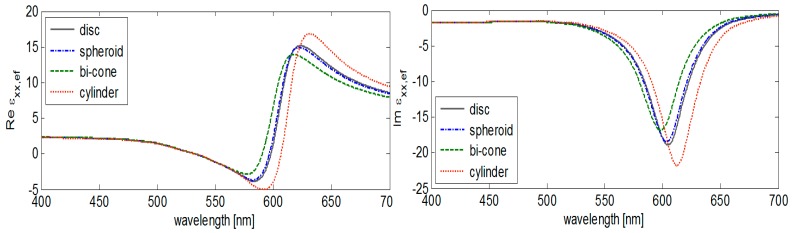
Shape dependent effective permittivity of system Au/polyacrylate (height/diameter = 10/5 nm, fill factor 0.2) for typical forms of nanoparticles.

The electric dipoles induced in metal nanoparticles by external fields strongly modify the optical function of plasmonic materials (e.g., Au, Ag) that is in the visible optical region manifested through the resonance peaks of the effective permittivity. In the Figure 4, we observe this effect close to the wavelength corresponding to the localized plasmon excitation [21]. The optical function of gold and the data for permittivity off-diagonal terms are adopted from References [22,23].

**Figure 5:**
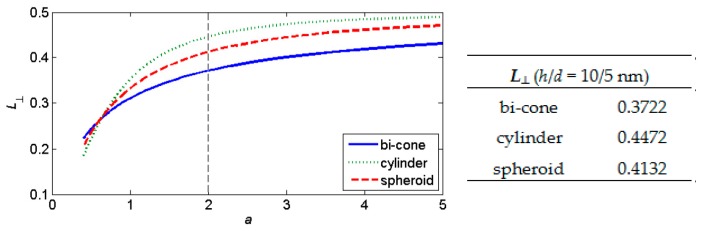
Depolarizing factor *L*_⊥_ dependence on the parameter *a*.

Effective permittivity dependence on depolarizing factors demonstrated in Figure 4 can be explained by a detailed analysis of Formulas (1) and (2). Simultaneously, we demonstrate shape-dependent effective permittivity of heterogeneous nanostructures containing inclusions of several forms. The cylindrical nanoparticles exhibit the most expressive effects corresponding to the depolarizing factors of this inclusions shape (see Figure 5).

The components of depolarizing tensor **L** are of the order tenths (Table 3). An affection of the generalized depolarizing Green tensor is:

(12) $$\langle G\rangle =-\frac{1}{k^{2}}\left[L-k^{2}M\right]=-\frac{1}{k^{2}}N$$

The correction term *k*^2^**M** is more significant for prolate nanoparticles having its diameter *d* less than the height *h* even as the particle height has prevailing effect. This fact is demonstrated by the data in Table 3 for cylinder or conical nanoparticles.

**Figure 6:**
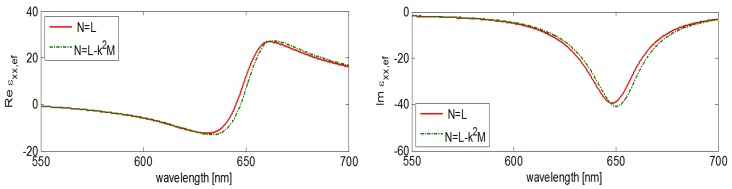
An influence of correction factor *k*^2^*M*_⊥_ on the real and imaginary part of effective permittivity component ε*_xx_*_,*ef*_: cylindrical Au nanoparticles in polyacrylate (height/diameter = 50/20 nm, fill factor 0.2).

Note that the resulting forms in Table 2 contain the particle radius *R* besides the characteristic parameter *a* that need be respected in applications of these model results. Moreover, the *k*^2^*M*_⊥_ term also depends on the wavelength through the factor *k*. An example of the outstanding correction factor influence on the effective permittivity in particular case (see the value emphasized in the Table 3) is demonstrated in the following figure (Figure 6).

## 4. Conclusions

The introduced extension of EMA via the SCD model for anisotropic nanoparticles offers a possible tool to analyze anisotropic nanostructured heterogeneous media in various applications, where natural or artificial composites (metamaterials) act as significant components of studied optical systems. As a novel aspect, an extension of the Green tensor regarding the volume-integral term is presented.

## Figures and Tables

**Table 1:** Depolarizing factors dependence on the geometric shape of nanoparticles.

Geometric Shape	Depolarizing Factor
CYLINDER	$L_{⊥}=\frac{1}{2}\frac{a}{\sqrt{1+a^{2}}}\;$
SPHEROID	$L_{⊥}=\frac{1}{2}\frac{a}{1-a^{2}}\;\{\begin{array}{l} \frac{\arccos a}{\sqrt{1-a^{2}}}-a,\;\mathrm{oblate}\;\\ \frac{1}{a}-\frac{a^{2}}{\sqrt{1-a^{2}}}\ln\frac{1+\sqrt{1-a^{2}}}{a},\;\left(a=\frac{d}{h}\right)\;\mathrm{prolate}\; \end{array}$
BI-CONE	$L_{⊥}=\frac{1}{2}\cdot \frac{a}{1+a^{2}}\left[a-1+\frac{1}{\sqrt{1+a^{2}}}\ln\frac{a}{\left(\sqrt{1+a^{2}}-a\right)\cdot \left(\sqrt{1+a^{2}}-1\right)}\right]$

**Table 2:** Correction factors dependence on the geometric shape of nanoparticles.

Geometric Shape	Correction Factors
CYLINDER	$M_{⊥}=-\frac{1}{2}R^{2}\ln\left(\sqrt{1+a^{2}}-a\right)\;,\;M_{∥}=R^{2}a\left(\sqrt{1+a^{2}}-a\right)$
BI-CONE	$M_{⊥}=\frac{aR^{2}}{4\sqrt{1+a^{2}}},\;M_{∥}=\frac{aR^{2}\left(1+2a^{2}-2a\sqrt{1+a^{2}}\right)}{2\sqrt{1+a^{2}}}$

**Table 3:** Correction factor *k*^2^*M*_⊥_ dependence on nanoparticle dimensions for cylinder (the first row) and bi-cone at fixed wavelength λ = 632 nm.

*k* ^2^ *M*_⊥_		*d* [nm]			
**CYLINDER/BI-CONE**		5	10	20	50
***h* [nm]**	5	0.0011/0.0002	0.0006/0.0006	0.0003/0.0012	0.0001/0.0031
	10	0.0071/0.0003	0.0044/0.0009	0.0024/0.0022	0.0010/0.0061
	20	0.0414/0.0003	0.0285/0.0011	0.0174/0.0035	0.0077/0.0115
	50	0.3704/0.0003	0.2857/0.0012	0.2035/0.0046	0.1089/0.0218
